# Continuous phenotypic modulation explains male horn allometry in three dung beetle species

**DOI:** 10.1038/s41598-022-12854-6

**Published:** 2022-05-24

**Authors:** Alex Laini, Angela Roggero, Claudia Palestrini, Antonio Rolando

**Affiliations:** Department of Life Sciences and Systems Biology, University of Turin, Via Accademia Albertina 13, 10123 Turin, Italy

**Keywords:** Entomology, Zoology, Software, Classification and taxonomy

## Abstract

Many dung beetle species show male horn polyphenism, the ability of males to develop into distinct phenotypes without intermediate forms as a response to the larval growth environment. While males with long (majors) and rudimentary (minor) horn have been widely reported in literature, little is known about the existence of individuals with intermediate horn length. Here we investigate the occurrence of intermediates in natural populations of three dung beetle species (*Onthophagus furcatus*, *Copris lunaris* and *C. hispanus*). We analysed the body size-horn length relationship using linear, exponential, and sigmoidal models with different error structures. We inferred the number of individuals in the minor, intermediate, and major groups by combining changepoint analysis and simulation from fitted allometric models. The sigmoidal equation was a better descriptor of the body size-horn length relationship than linear or exponential equations in all the three studied species. Our results indicated that the number of intermediates equals or exceeds the number of minor and major males. This work provides evidence that, at least in the studied species, males with intermediate horn length exist in natural populations. For similar cases we therefore suggest that continuous phenotypic modulation rather than discrete polyphenism can explain variation in male horn allometry.

## Introduction

Polyphenism is the ability of individual genotypes to develop into distinct phenotypes without intermediate forms in response to environmental changes^1,2^. Polyphenisms are modelled as threshold traits in which individuals below and above a “critical threshold” develop into different distinct morphologies^3^. They involve a size-dependent reprogramming event that alters larval development^4–6^, as opposed to a continuous phenotypic modulation that causes continuous variation in growth parameters^7,8^.

Horned dung beetles have traditionally been considered polyphenic because of a discontinuity in the scaling relationship between body size and horn length. Pioneering works on *Onthophagus acuminatus* (Harold, 1880) and *O. taurus* (Schreber, 1759) suggested a bimodal distribution of the cephalic horn length in males with individuals larger than a critical body size developing long horns on their heads (majors), whereas the others develop only rudimentary horns (minors)^2,9,10^. The transition between the two phenotypes was attributed to the characteristics of the larval growth environment, especially by the amount and quality of food received during the larval stage^9,10^ but also by population density^11^ and temperature^12^. Following these findings, the presence of two male morphs has proved to be widespread and consistent among horned dung beetles. Male dimorphism is usually inferred from the sigmoidal relationship with steep slopes existing between body size and horn length^2,9,10,13,14^. The switch point of the sigmoidal equation represents the “critical” body size that separates short- from long-horned individuals. The steep slope guarantees that the transition between short- and long-horned individuals will abruptly occur within a small body size range^15^. The sigmoidal relationship results in a bimodal distribution of horn lengths, in which intermediate forms are rare compared to minor and major individuals.

The distinction between minor and major individuals is also supported by differences in mating strategies between the two morphs^9,10^. In some species of the genus *Onthophagus*, large-horned individuals use their horns as weapons in fights to provide and defend access to females inside tunnels. Small individuals adopt a sneaking tactic by digging alternative tunnels or utilising unguarded tunnels to avoid large males. Mating strategies are partially driven by allocation trade-offs among traits, where horns compete with other traits for common growth factors. A trade-off between horn length and testes size has been found, with small individuals allocating more energy to testes growth than large individuals in several *Onthophagus*^16^. Similar trade-offs have been reported for the dimension of eyes, legs, antennae, and other morphological traits^17–19^. Individuals with an intermediate horn length are thus competitively disfavoured because they perform both reproductive tactics poorly and due to intermediate physiological adaptations^20^.

In line with allometric and behavioural expectations, most studies have found a paucity of intermediate forms in natural dung beetle populations. However, evidence that individuals with intermediate horn length^15,21^ and that the allometric relationships may be framed in a continuous phenotypic modulation^8^ exist, challenging the entire idea that polyphenism is widespread and consistent in horned dung beetles. Here we studied the body size-horn length relationship in three dung beetle species (*Copris lunaris* (Linnaeus, 1758), *Onthophagus furcatus* (Fabricius, 1781), *Copris hispanus* (Linnaeus, 1764) with a differing slope of the sigmoid by combining changepoint analysis and simulations from fitted allometric models to identify the number of individuals in the minor, intermediate, and major groups. We use our findings to discuss the mechanisms regulating the body size-horn length relationship and the implications for mating strategies.

## Results

**Figure 1:**
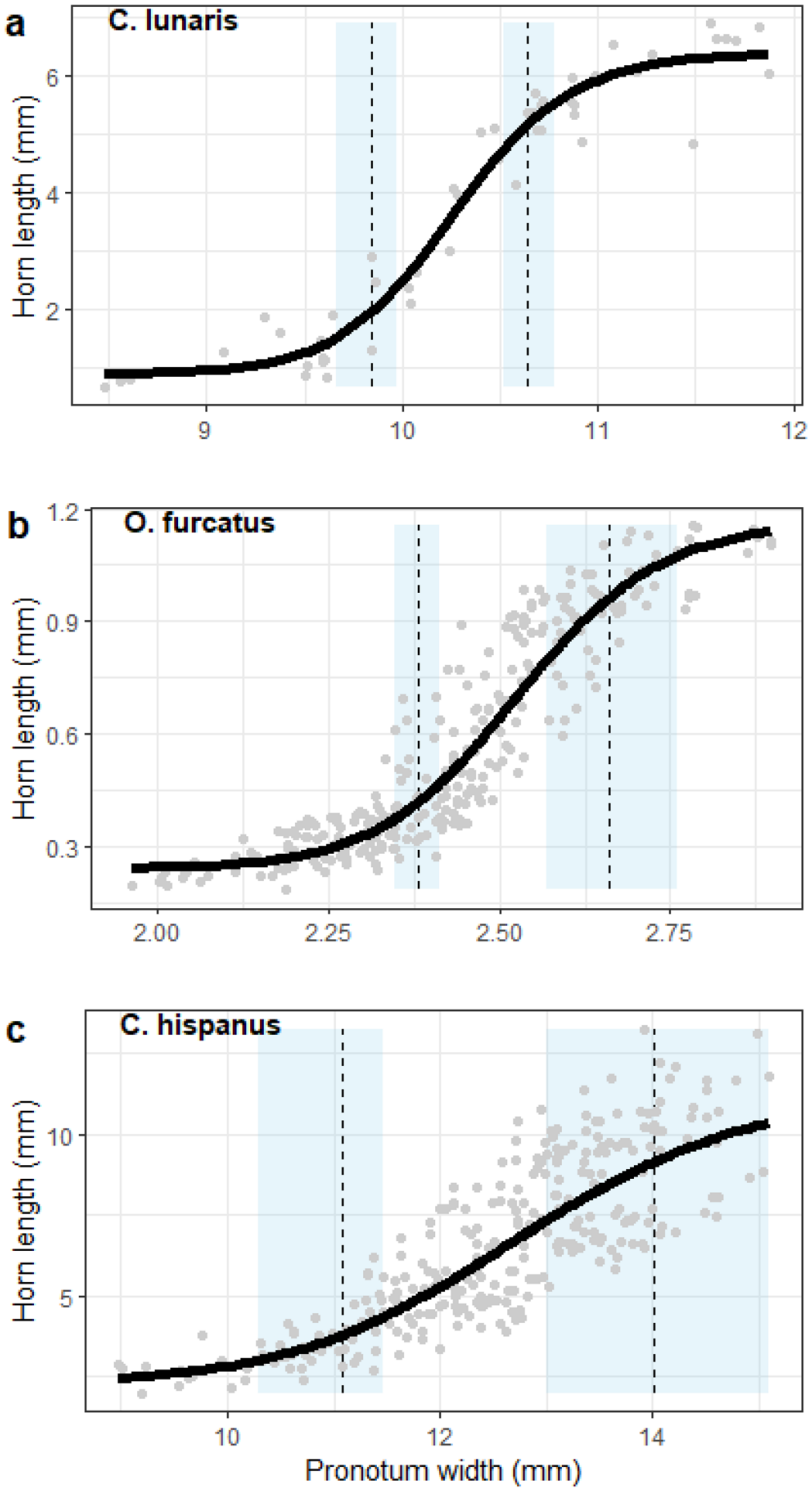
The body size-horn length relationship for Copris lunaris, Onthophagus furcatus, and Copris hispanus. The solid black line is the best-fitted line according to a sigmoidal equation. The dashed lines and the light blue shaded areas represent the median and confidence interval of the changepoints between the minor-intermediate (on the left) and the intermediate-major groups (on the right).

We analysed the body size-horn length relationship in natural populations of *C. lunaris*, *O. furcatus* and *C. hispanus*. We tested this relationship using linear, exponential, and sigmoidal models with different error structures. The best-fitted model was sigmoidal with additive normal homoscedastic errors for *C. lunaris* and sigmoidal with multiplicative lognormal heteroscedastic errors for *O. furcatus* and *C. hispanus* (Fig. 1, Supplementary 1). Slope parameter b in the sigmoidal equation was 34.1, 24.1 and 11.5 for *C. lunaris*, *O. furcatus,* and *C. hispanus*, respectively. No apparent discontinuities were found by visual inspection or in log–log plots of the body size-horn length relationship (Supplementary 2).

**Figure 2:**
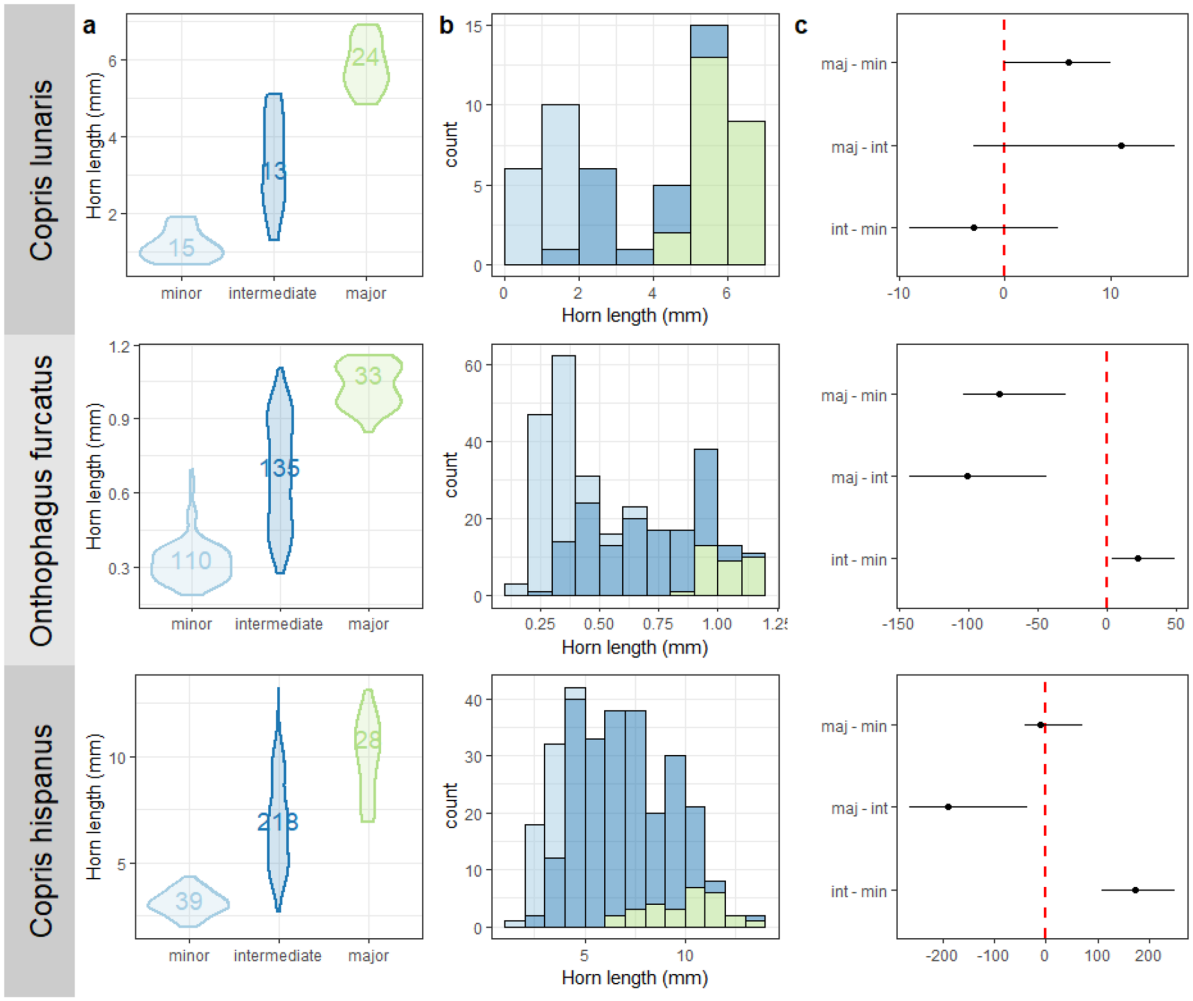
Occurrence of minor, intermediate, and major individuals in three dung beetle species. (**a**) Violin plot of the horn length of the minor (light blue), intermediate (blue), and major (green) groups for three dung beetle species. The number of individuals in each group is also reported; (**b**) histograms of the horn length divided by group; (**c**) confidence intervals of the difference among the number of individuals in the minor, intermediate, and minor groups. The median values (black circles) and 95% percentile intervals (solid lines) of differences are reported. The dashed red lines indicate 0.

We performed a changepoint analysis to identify the number of individuals in minor, intermediate, and major groups. The number of individuals and the body size range (i.e. the difference between maximum and minimum body size) of the intermediate group differed among species (Fig. 2a). *Copris lunaris* had the fewest individuals (median = 13, 25% of the analysed individuals) and the narrowest body size range of the intermediate group (median = 0.79 mm, 23% of the overall body size range). The intermediate group of *O. furcatus* was composed of 133 individuals (48%) with a body size range of 0.27 mm (29%), while that of *C. hispanus* contained 218 individuals (76.5%) with a body size range of 2.9 mm (47.4%). In *O. furcatus*, the number of observed and simulated individuals in the intermediate group were due to variability introduced with random number generation. The analysed species differed in the horn length dimorphism, with *C. lunaris*, *O. furcatus*, and *C. hispanus* having marked bimodal, weak bimodal, and unimodal distributions, respectively. The recognition of intermediates resulted in less marked bimodal distribution peaks for minors and majors, especially for the majors in *O. furcatus* (Fig. 2b).

We determined the significance of the difference in the number of individuals in minor, intermediate, and major groups by simulating the regression parameters values from the variance–covariance matrix of the parameters using multivariate normal distribution. The number of individuals in the minor, intermediate, and major groups did not significantly differ in *C. lunaris* (confidence intervals completely overlapped 0; Fig. 2c). Intermediates outnumbered the minors and majors in both *O. furcatus* and *C. hispanus*, while minors were more numerous than majors only in *O. furcatus*.

## Discussion

Horn length polyphenism in dung beetles has fascinated generations of scientists and continues to lead to debate about its underlying mechanisms. A separation into two distinct morphs with rudimentary (minor) and fully developed (major) horns is typically reported for most dung beetle species. By using allometric modelling coupled with a changepoint analysis and simulation from fitted models, we demonstrated that the number of intermediates equals or exceeds those of the minors and majors in three species differing for the steepness of the body size-horn length relationship.

The sigmoidal equation was a better descriptor of the body size-horn length relationship than linear or exponential equations in the three studied species. The slope of the sigmoid regulates the abruptness of the transition between minors and majors that, in turn, determines the body size range and the number of individuals with intermediate horn length. Although an increased slope results in a narrow body size range of the intermediates in *C. lunaris*, the number of individuals with intermediate horn length equals those of minors and majors. Thus, the sigmoid slope per se does not evidence polyphenism because it allows for the presence of intermediates. Moreover, it does not inform analysts about the presence of discontinuities in the body size-horn length relationship because sigmoids represent, de facto, continuous relationships.

Our approach to identify changepoints relied on sigmoidal equation parameters and consistently recognises many intermediates in the three studied dung beetle species. We found that a bimodal horn length distribution does not necessarily translate into more individuals showing either rudimentary or fully developed horns. Classifying individuals into morphs is a major challenge given the inherent random variability in both horn length and body size. For example, individuals with different body sizes can have the same horn length, and vice versa. Alternative approaches to those herein followed to classify individuals into morphs exist but are often flawed and none is superior to others in all situations^22^. This is probably because the identification of discrete morphs is somewhat subjective given the choice of criteria to establish different categories^23^. In broader terms, the entire morphs classification process in most dung beetle species appears inaccurate because of the attempt to discretise a continuous relationship with no apparent discontinuities. We thus raise questions about the validity of the discontinuous allometric variation concept in dung beetles, at least in some species. Similar conclusions have been drawn by Packard (2021)^24^ when analysing the body size-horn length relationship in rhinoceros beetles, which is another group of polyphenic beetles that bear cephalic horns.

Two alternative developmental mechanisms can explain the emergence of sigmoidal relationships: reprogramming and extreme positive allometries^25^. Reprogramming involves the presence of critical thresholds that alter larvae development^4^ and can emerge when two components (e.g., hormones) mutually exclude one another^6,7^. Alternatively, horn length polyphenism can be explained by continuous phenotypic modulation and, therefore, by extreme positive allometries (when the trait has a higher growth rate than the body as a whole) until resources are exhausted^8,25^. The overall process of continuous phenotypic modulation is regulated by sharp transitions in the gene activities triggered by the amount of dung available for larval growth^7,25^. According to Tomkins et al. (2006)^8^, our results are more consistent with the mechanism of continuous phenotypic modulation rather than threshold reprogramming because we did not find discontinuities in the body size-horn length relationship and a rather large number of individuals with intermediate forms appears in all three studied species. This view is supported by recent transcriptomic screens and gene function assays, which reveal that changes in expression rather than in the type of regulating genes determine horn length in dung beetles^27^.

The recognition of intermediates in natural populations of dung beetles poses problems for interpreting their mating behaviour because intermediates are expected to perform poorly compared to minors and majors. Hornless individuals continue to display aggressive behaviour and engage in fights with other hornless males, and even with horned males, although the probability of success in the latter is virtually null^20^. Hence intermediates can adopt opportunistic behaviour by engaging in fights with hornless or similar sized individuals, using sneaking tactics or waiting for unguarded females when facing larger individuals. However, mating tactics are only one of the variables that affect the mating success probability in natural populations given the effect of multiple co-occurring factors. Body size, horn length, and strength contribute to the probability of winning fights in staged contests of *O. taurus*^28,29^. This makes the outcome of fights difficult to predict when these features take contrasting patterns in two contestants (e.g., long-horned individuals with a smaller body size vs. short-horned individuals with a larger body size). Density-dependent processes, including asymmetric competition with larger species^30^, can affect the probability of accessing resources and thus favour the selection for specific body sizes. Dung age^31^, courtship rate^29^, and interspecific differences in horn use as competitive signals^32^ are other variables that co-occur to determine an individual’s mating success and may justify the occurrence of intermediate forms.

Our work provides evidence that individuals with intermediate horn length exist in natural populations of some dung beetle species and discusses the implications of regulating mechanisms and mating tactics. We suggest for at least some dung beetle species with allometric body size-horn length relationships can be better framed in a continuous phenotypic modulation context than discrete polyphenism, and minors and majors represent the extremes of underlying continuous distribution.

## Methods

### Horn length and body size

We used 52, 278, and 285 individuals of *C. lunaris*, *O. furcatus* and *C. hispanus* collected from the field. *Copris hispanus* and *C. lunaris* individuals were collected entirely from natural populations in Italy, while *O. furcatus* individuals were collected from natural populations in both Italy (n = 102) and Turkey (n = 176). We acquired the cephalic weaponry and pronotum images with a Leica® DMC4500 (Leica Microsystems AG, Wetzler, Germany) digital camera connected to a Leica® Z16APO stereoscopic dissecting microscope (Leica Microsystems AG, Wetzler, Germany). The LAS-Leica Application Suite software (Leica Microsystems AG, Wetzler, Germany) was used to capture and store photographs, and to measure the horn length and pronotum maximum width, a reliable index of body size for coleopterans^22^. Horn length of *C. lunaris* and *C. hispanus* was measured as a curved line from the base to the tip of the horn^33^, and as a straight line from the base to the tip for *O. furcatus*.

### Regression models

We investigated the body size-horn length relationship using linear general form power and sigmoid equations. Three error structures were investigated for each function: additive normal homoscedastic, additive normal heteroscedastic, and multiplicative lognormal heteroscedastic^24^. We selected the best model according to the Akaike Information Criterion (AIC) among the nine models that emerged from the combinations between equations and error structure. Computations were performed using the Model Procedure in SAS 9.4.

### Changepoint analysis

We predicted horn length from the best-fitted model on 1,000 points equally spaced within the interval between the minimum and maximum measured body sizes. We calculated the slope for each point by the finite difference method. Then the difference between the slope of a point and that of the previous one was calculated. The biggest and smallest differences were taken as the changepoints between the minor-intermediate and intermediate-major groups, respectively. These changepoints were then used to calculate the number of individuals in each group. We evaluated the uncertainty of changepoints by simulating 1,000 regression parameters values from the variance–covariance matrix of the parameters using multivariate normal distribution^34^. We performed the changepoint analysis for each random draw of the parameter. The confidence interval of the changepoint and the associated measures were then determined as the percentile interval (2.5–97.5%). We also calculated the pairwise differences among the number of individuals of the minor, intermediate, and major groups for each species from the simulated values. For *O. furcatus*, the individuals from different countries were pooled because they provided similar results (Supplementary 3). The changepoint and uncertainty analyses were performed with the R statistical software^35^.

## Supplementary Information

Supplementary Information 1.

Supplementary Information 2.

## Data Availability

Source data to generate figures and tables are available from the corresponding authors.
