# Supplementary material for: Continuous phenotypic modulation explains male horn allometry in three dung beetle species
Source: Sci Rep. 2022 May 24;12:8691. doi: 10.1038/s41598-022-12854-6 (PMC9130230; doi:10.1038/s41598-022-12854-6)
Supplement: Supplementary file 1 — Supplementary Information 1. [file 41598_2022_12854_MOESM1_ESM.docx]

**# SYSTEM REQUIRMENTS**

**# R version 4.0.3 (2020-10-10) -- "Bunny-Wunnies Freak Out" or higher**

**# R packages and version used. Most updated versions of these packages should also work.**

**# MASS_7.3-53**

**# ggplot2_3.3.3**

**# tidyverse_1.3.0**

**# readxl_1.3.1**

**# The function was tested on Windows 10 x64 (build 19042), but it should work on any operating system.**

**#**

**# INSTALLATION GUIDE**

**# A working installation of R and the required packages is everything you need.**

**# If packages are missing install them as usual, for example from the command line:**

**#**

**# install.packages("ggplot2")**

**# FUNCTION FOR CALCULATING SWITCHPOINTS**

**# intermediate_count_uncertainty: a function for calculating the number of minor, intermediate and major**

**# individuals models that provide uncertainty estimates by simulation from fitted allometric models**

**#**

**# author: Alex Laini**

**#**

**# data_orig: a data.frame with body size and horn length observations.**

**# v_cov: variance covariance matrix of the estimated parameters.**

**# n: number of simulations.**

**# eps: a small amount for estimating slopes with the finite difference method.**

**# length.out: number of points used for calculating slope.**

**#**

**# this function provide a list of length 3:**

**# simulated_coefficients: a data.frame listing all the simulated coefficients.**

**# summary_results: a data.frame containing summary statistics on the minor, intermediate and major groups**

**# switchpoints: list the lower and higher switchpoints for all the simulation.**

intermediate_count_uncertainty <- function(data_orig, v_cov, n = 10, eps = 10^-7, length.out = 100){

require(MASS)

mean_cl <- v_cov[1, ] # parameter estimates

vc <- v_cov[-1,]

coeff <- t(mvrnorm(n, mean_cl, vc))

# sigmoidal function

cl_function <- function(x) yzero + (a * x^b) / (x^b + xzero^b)

res_min <- res_max <- c()

x <- data_orig$body_size

for(i in 1:n){

a <- coeff[2, i]

b <- coeff[3, i]

xzero <- coeff[1, i]

yzero <- coeff[4, i]

x_values <- seq(min(x), max(x), length.out = length.out)

x_orig <- cl_function(x_values)

x_eps <- cl_function(x_values + eps)

x_diff <- (x_eps - x_orig) /eps

x_diff_slopes <- x_diff[-1] - x_diff[-length(x_diff)]

res_temp_min <- (x_values[which.max(x_diff_slopes)+1] + x_values[which.max(x_diff_slopes)])/2

res_temp_max <- (x_values[which.min(x_diff_slopes)+1] + x_values[which.min(x_diff_slopes)])/2

res_min <- c(res_min, res_temp_min)

res_max <- c(res_max, res_temp_max)

}

group_min <- sapply(res_min, function(z) sum(x <= z))

group_max <- sapply(res_max, function(z) sum(x >= z))

group_int <- rep(length(x), n) - group_min - group_max

res_body_min <- quantile(res_min, c(0.025, 0.5, 0.975))

res_body_max <- quantile(res_max, c(0.025, 0.5, 0.975))

res_body_diff <- quantile(res_max - res_min, c(0.025, 0.5, 0.975))

res_min_def <- quantile(group_min, c(0.025, 0.5, 0.975))

res_max_def <- quantile(group_max, c(0.025, 0.5, 0.975))

res_intermediate_def <- quantile(group_int, c(0.025, 0.5, 0.975))

res_minor_int_diff <- quantile(group_int - group_min, c(0.025, 0.5, 0.975))

res_major_int_diff <- quantile(group_max - group_int, c(0.025, 0.5, 0.975))

res_major_minor_diff <- quantile(group_max - group_min, c(0.025, 0.5, 0.975))

res <- rbind(res_body_min, res_body_max, res_body_diff, res_min_def, res_max_def, res_intermediate_def, res_minor_int_diff, res_major_int_diff, res_major_minor_diff)

results <- list(coeff, res, data.frame(res_min, res_max))

names(results) <- c("simulated_coefficients", "summary_results", "switchpoints")

results

}

**# WORKED EXAMPLE**

library(readxl)

library(tidyverse)

library(MASS)

library(ggplot2)

# Copris lunaris data

c_lunaris <- data.frame(body_size = c(10.399, 10.978, 10.469, 10.258, 10.277, 10.71, 10.718, 10.642, 10.875, 10.918, 10.685, 11.073, 11.274, 11.708, 11.577, 10.853, 10.879, 10.486, 11.196, 10.984, 10.709, 10.669, 11.823, 11.484, 10.682, 10.868, 11.875, 11.6, 10.578, 11.655, 10.027, 9.585, 9.858, 9.595, 9.609, 9.647, 10.067, 9.293, 9.084, 10.237, 10.04, 9.594, 9.503, 9.608, 9.057, 9.372, 8.612, 8.559, 8.481, 9.516, 9.843, 9.844),

horn_length = c(5.042, 5.936, 5.087, 4.052, 3.979, 5.076, 5.562, 5.374, 5.329, 4.874, 5.07, 6.522, 6.372, 6.614, 6.899, 5.552, 5.513, 4.578, 6.052, 5.985, 5.484, 5.358, 6.844, 4.847, 5.717, 5.983, 6.03, 6.632, 4.127, 6.627, 2.371, 1.445, 2.474, 1.162, 0.814, 1.899, 2.619, 1.848, 1.268, 2.981, 2.105, 1.094, 0.847, 1.135, 0.941, 1.6, 0.803, 0.747, 0.651, 1.017, 1.276, 2.909)) %>%

as_tibble()

vcov_c_lunaris <- matrix(c(10.25921199602, 0.00195295214659227, -0.000191275652283669, 0.00771333304405531, 0.00391096069507193, 5.53465475289677, -0.000191275652283669, 0.0917148237089207, -1.12402753994161, -0.045318676417689, 34.1357517757448, 0.00771333304405531, -1.12402753994161, 20.1110621333974, 0.539086420358642, 0.867563523415351, 0.00391096069507193, -0.045318676417689, 0.539086420358642, 0.0340491069442123), ncol = 4)

colnames(vcov_c_lunaris) <- c("x0", "a", "b", "y0")

# set the number of simulation

N <- 1000

# perform the analysis

c_lunaris_un <- intermediate_count_uncertainty(c_lunaris, vcov_c_lunaris, n = N, length.out = 1000)

### plot sigmoids obtained by simulation from fitted allometric models ###

# sigmoid function used in this work

sigmoid_func <- function(x,y) y[4] + (y[2] * x^y[3]) / (x^y[3] + y[1]^y[3])

res_simu <- as.data.frame(apply(c_lunaris_un[[1]], 2, function(z) sigmoid_func(c_lunaris$body_size, z)))

colnames(res_simu) <- paste("simu", 1:N, sep = "_")

res_simu <- data.frame(c_lunaris[, "body_size"], res_simu)

res_simu_long <- res_simu %>%

pivot_longer(-body_size)

ggplot(res_simu_long, aes(x = body_size, y = value, fill = name)) +

geom_line(alpha = 0.1, col = "grey80") +

theme_bw() +

theme(legend.position = "none") +

geom_point(data = c_lunaris, aes(x = body_size, y = horn_length), inherit.aes = FALSE) +

labs(x = "Body size (mm)", y = "Horn length(mm)")
