# Supplementary material for: Continuous phenotypic modulation explains male horn allometry in three dung beetle species
Source: Sci Rep. 2022 May 24;12:8691. doi: 10.1038/s41598-022-12854-6 (PMC9130230; doi:10.1038/s41598-022-12854-6)

Supplementary Information 1

Log-likelihood and AIC for the relationship between body size and horn length analyzed with linear, exponential and sigmoidal models with different error structures for the *Copris lunaris*, *Onthophagus furcatus* and *Copris hispanus*. General form of linear, power and sigmoid equations were $y = a + xb$, $y = ax^b$ and $y = y_0 + \frac{ax^b}{x^b + x_0^b}$, respectively.

Copris lunaris

**Model**	**LogLik**	**AIC**	**ΔAIC**
linear – anho	59.7	125.5	48.5
linear – anhe	-59.6	127.1	50.1
linear – mlhe	-80.3	166.7	89.7
power – anho	-59.7	127.4	50.4
power – anhe	-59.5	129.8	52.8
power – mlhe	-67.2	142.4	65.4
sigmoid 4 - anho	-33.5	77.0	0.0
sigmoid 4 - anhe	-32.7	77.5	0.5
sigmoid_4 - mlhe	-47.9	105.7	28.7

Onthophagus furcatus

**Model**	**LogLik**	**AIC**	**ΔAIC**
linear – anho	180.6	-355.2	152.1
linear – anhe	180.7	-353.4	153.9
linear – mlhe	160.4	-314.8	192.5

power – anho	195.5	-382.9	124.4
power – anhe	225.8	-441.6	65.7
power – mlhe	144.9	-281.8	225.5
sigmoid – anho	241.4	-472.7	34.6
sigmoid – anhe	254.6	-497.2	10.1
sigmoid – mlhe	258.7	-507.3	0.0

Copris hispanus

Model	LogLik	AIC	ΔAIC
linear – anho	-515.8	1037.6	72.6
linear – anhe	-504.4	1016.8	51.8
linear – mlhe	-504.2	1014.4	49.4
power - anho	-513.3	1034.5	69.5
power - anhe	-489.7	989.4	24.4
power - mlhe	-484.1	974.2	9.2
sigmoid - anho	-508.8	1027.5	62.5
sigmoid - anhe	-481.6	975.2	10.2
sigmoid - mlhe	-477.5	965.0	0.0

Supplementary Information 2

Log-log plot of body size versus horn length for *Copris lunaris*, *Onthophagus furcatus* and *Copris hispanus*, where ΔX = ΔY. No apparent discontinuities are present, showing the continuous nature of the relationship between body size and horn length.

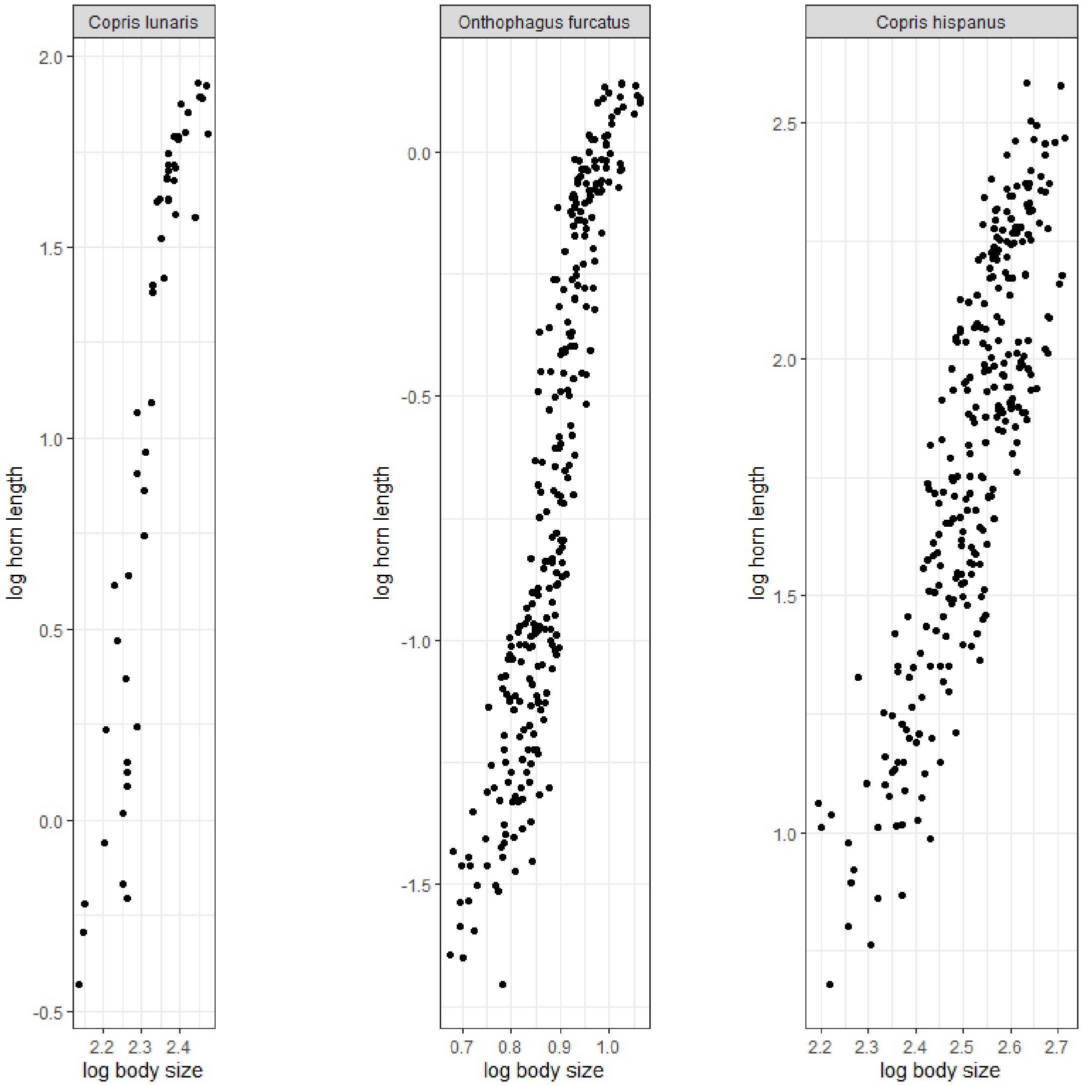

Supplementary Information 3

Copris lunaris
Individuals of *C. lunaris* were collected by the authors in northern Italy (La Mandria natural Park) and obtained on loan from the Museo Civico di Storia Naturale, Milano, Italy (MSNM) and the Museo Zoologico “La Specola”, Firenze, Italy (MZUF).

Copris hispanus
Individuals of *C. hispanus* were obtained on loan from the Museo Zoologico “La Specola”, Firenze, Italy (MZUF). The geographical range of the available individuals spans from southern Italy to Spain.

Onthophagus furcatus
O. furcatus individuals were collected from natural populations in both northern Italy (n=102, several localities representing the entire longitudinal range of the Po plain) and Turkey (n=176, several localities near Amasya).

Individuals collected by the authors are stored at the University of Torino, Department of Life Sciences and Systems Biology, Torino (MIZT).

Supplementary Information 4

Log-log plot of body size versus horn length for *Onthophagus furcatus* collected in Italy and in Turkey, where ΔX = ΔY. No apparent discontinuities are present, showing the continuous nature of the relationship between body size and horn length.

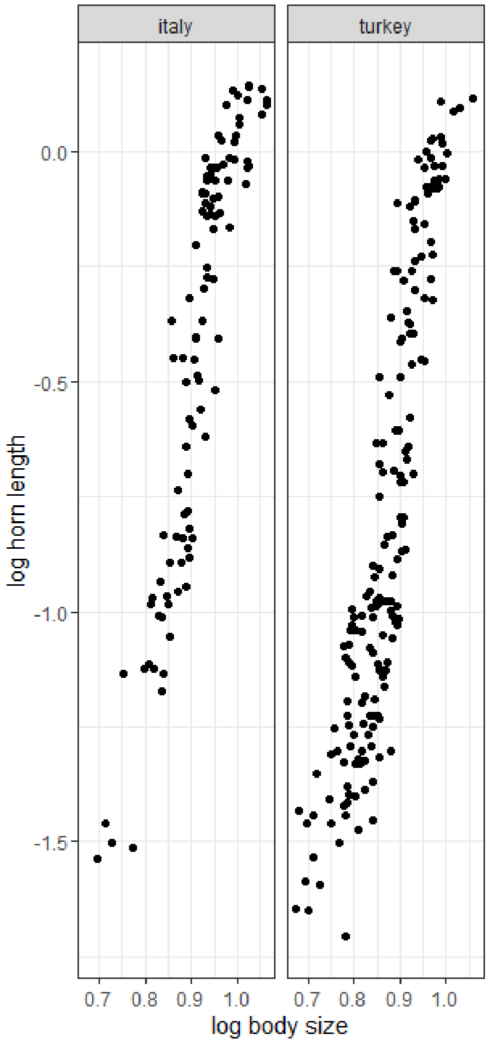

Supplement: Supplementary file 2 — Supplementary Information 2. [file 41598_2022_12854_MOESM2_ESM.pdf]
